# Impact of Medication Dose Optimization on Heart Failure Outcomes in African-American Female Patients: A Safety-Net Hospital Experience

**DOI:** 10.7759/cureus.87443

**Published:** 2025-07-07

**Authors:** Ricardo A Rodriguez Mejia, Eric Acker, Tark Abou-elmgd, Thirumala Keerthi Chandrika Kammaripalle, Humza Rana

**Affiliations:** 1 Internal Medicine, Cape Fear Valley Medical Center, Fayetteville, USA

**Keywords:** african american women, comorbidities, heart failure, medication prioritization, safety-net hospital

## Abstract

Heart failure among African-American female patients represents a significant public health challenge, with hospitalization rates being much higher than those of White female patients. The high prevalence of comorbidities in this population often necessitates the selective implementation of guideline-directed medical therapy (GDMT). This study examines which medication classes should be prioritized to improve outcomes in this vulnerable population.

We conducted a retrospective study of 283 African-American female patients with heart failure admitted to Cape Fear Valley Medical Center, a safety-net hospital serving low- to medium-income patients in North Carolina, between 2021 and 2022. We analyzed the relationships between GDMT regimens and clinical outcomes using multivariable logistic regression. A GDMT composite, ranging from zero to nine, was developed to measure overall medication optimization.

Among the 283 patients studied, 141 (50%) experienced a 30-day readmission, 161 (57%) a 90-day readmission, and 65 (23%) died within a year. Medication utilization was suboptimal: 28 patients (10%) received the goal doses of renin-angiotensin system (RAS) inhibitors, 37 (13%) were administered goal doses of beta blockers, 11 (4%) received medium/goal doses of mineralocorticoid receptor antagonists (MRAs), and 31 (11%) were given sodium-glucose cotransporter 2 (SGLT2) inhibitors at goal doses. The mean GDMT composite was 2.4±1.8, with only 23 patients (8%) achieving a composite of greater than or equal to five. Each one-point increase in GDMT composite reduced 30-day (OR=0.85, p=0.02) and 90-day (OR=0.86, p=0.03) readmission risks. A higher GDMT composite was associated with decreased mortality in the unadjusted analysis (OR=0.86, p=0.07), with mortality rates declining from 28% (11/39) with a GDMT composite of zero to 10% (1/10) with a composite of greater than or equal to eight. Concurrent optimization of RAS inhibitors and beta blockers reduced readmission risk (OR=0.70, p=0.04). Low-dose MRA lowered 30-day readmission (OR=0.27, p<0.01) and medium-dose beta blockers reduced one-year mortality (OR=0.13, p=0.03), as did medium doses of MRA (OR=0.01, p<0.01). Key clinical predictors included lower ejection fraction (OR=0.95, p<0.01), previous hospitalizations (OR=3.37, p<0.01), and chronic kidney disease (OR=2.49, p=0.03).

In a safety-net hospital setting, strategic prioritization of specific GDMT components improved outcomes among African-American female patients with heart failure and multiple comorbidities. Each one-point increase in the GDMT composite was associated with a 15% reduction in readmission risk and a trend toward lower mortality. Beta blockers should be prioritized for mortality reduction, MRAs for both mortality and readmission reduction, and RAS inhibitors with beta blockers for reducing readmissions. These findings inform medication strategies for clinicians serving similar vulnerable populations.

## Introduction

Heart failure (HF) among African-American women represents a significant public health crisis, with hospitalization rates 2.6 times higher than those of White women and 30-day readmission rates exceeding 25% [1]. This condition remains a major contributor to morbidity, mortality, and healthcare expenditures in the United States, with persistent disparities disproportionately affecting African-American populations [1]. Hospitalization rates for HF in Black individuals are nearly two-and-a-half times higher than those of White individuals, with significantly greater healthcare costs incurred within the first year following discharge [2,3]. Despite advances in HF management and modest improvements among other racial and ethnic groups, the disparity in HF-related outcomes between Black and White patients has remained largely unchanged over the past decade. This gap is driven by a multifaceted interplay of biologic susceptibility, social determinants of health, and systemic inequities in healthcare delivery [4,5].

Emerging evidence suggests that these disparities are even more pronounced in African-American female populations. National Health and Nutrition Examination Survey (NHANES) data reveal that Black women score significantly lower on Life's Simple 7 cardiovascular health metrics compared to White women, while racial differences among men are less prominent [6]. Despite higher rates of obesity and atherosclerotic cardiovascular disease (ASCVD) risk factors, Black women are less likely to attempt weight loss or report adherence to heart-healthy diets [7]. These risk factor disparities translate directly into treatment challenges, highlighting the need for focused research and intervention strategies tailored to this underrepresented, high-risk subgroup.

Guideline-directed medical therapy (GDMT) for HF with reduced ejection fraction (HFrEF) has revolutionized the its management, offering significant improvements in survival and quality of life [8,9]. Current guidelines recommend a four-pillar approach including beta-blockers, renin-angiotensin system (RAS) inhibitors, mineralocorticoid receptor antagonists (MRAs), and sodium-glucose cotransporter 2 (SGLT2) inhibitors for optimal management. However, equitable implementation of GDMT including appropriate prescribing, dose titration, and adherence, remains a challenge [10,11]. While Eberly et al. documented SGLT2 inhibitor disparities using national claims data [12] and the Change the Management of Patients with Heart Failure (CHAMP-HF) Registry found only 1% of HFrEF patients achieved target doses of all GDMT medications [13], neither study addressed the key question for safety-net providers: which medications can be prioritized when serving African-American female patients with multiple comorbidities that preclude full GDMT optimization. Our study fills this gap by analyzing real-world outcomes in 283 African-American female patients where complete GDMT implementation was achieved in only 23 (8%) patients.

The high prevalence of comorbidities in African-American female patients with HF significantly complicates GDMT implementation [10,11]. With most patients in our cohort having hypertension (94%), chronic kidney disease (69%), diabetes (65%), and coronary artery disease (59%), medication contraindications and interactions often necessitated selective implementation of GDMT components [14]. Limited data exists to guide clinicians on which elements of quadruple therapy should be prioritized for this population [12,13]. Our study addresses this critical gap by examining the relative benefits of individual medication classes, aiming to inform more targeted and effective treatment approaches for this high-risk group with complex care needs.

To address this knowledge gap, we conducted a retrospective observational study examining the relationship between GDMT regimens and clinical outcomes among African-American female patients with HF admitted to Cape Fear Valley Medical Center between January 2021 and December 2022. Cape Fear Valley serves as a safety-net hospital for low- to medium-income patients throughout eastern North Carolina, with a diverse patient population that includes a substantial African-American community. This setting is particularly relevant as safety-net hospitals often face unique challenges in implementing complex medication regimens while serving vulnerable populations with limited resources. Our analysis focused on prescribing patterns, medication adherence, and clinical outcomes including readmission rates, disease progression, and mortality. Findings from this study may inform targeted interventions and policy changes to improve HF management and reduce disparities in this vulnerable population.

## Materials and methods

Study design and participants

We conducted a retrospective cohort study of African-American female patients hospitalized with HF at Cape Fear Valley Medical Center, a safety-net hospital in Fayetteville, North Carolina, between January 2021 and December 2022. We included self-identified African-American female participants aged ≥18 years with a primary or secondary diagnosis of acute decompensated HF who had complete documentation regarding their discharge medication, and at least 12 months of follow-up data. We excluded patients with reversible causes of HF, those discharged to a hospice or who died during hospitalization, and those with incomplete data. Of the 309 eligible patients, 26 were excluded for missing data, yielding 283 participants.

Data collection and outcomes

We extracted demographic data (age, insurance status, BMI, and smoking status), clinical characteristics (HF type, ejection fraction (EF), New York Heart Association class (NYHA), and comorbidities), and discharge medications from electronic medical records. Based on the EF, HF was classified as reduced (HFrEF, EF<40%), mid-range (HFmrEF, EF 40-49%), or preserved (HFpEF, EF≥50%). Comorbidities included hypertension, coronary artery disease, diabetes, chronic kidney disease, and atrial fibrillation, identified using International Classification of Diseases (ICD)-10 codes.

Our primary outcomes were 30-day and 90-day all-cause hospital readmission and one-year all-cause mortality. Readmissions were identified through our hospital system and regional health information exchange. Mortality was ascertained through hospital records, health information exchange, and Social Security Death Index queries.

Medication classification

We recorded discharge doses for all HF medications and classified them into four categories based on the percentage of the guideline-recommended target dose: none/contraindicated (0), low (1-33%), medium (34-66%), and goal (67-100%). For medications with fixed dosing (SGLT2 inhibitors, digoxin), we used binary coding. We created a composite RAS inhibitor variable representing the maximum dose among angiotensin converting enzyme (ACE) inhibitors, angiotensin receptor blockers (ARBs), or angiotensin receptor-neprilysin inhibitor (ARNI).

We developed three summary measures: Combo 1 (either RAS inhibitor or beta-blocker at goal dose), Combo 2 (at least one core medication at medium dose or higher), and a GDMT composite score (0-9) calculated by summing dose categories for RAS inhibitors (0-3), beta-blockers (0-3), MRAs (0-3), and SGLT2 inhibitors (0-1).

Statistical analysis

We summarized continuous variables as mean±SD or medians (IQR) based on distribution, and categorical variables as frequencies and percentages. We used chi-square tests for categorical comparisons and t-tests or ANOVA for continuous variables.

For each outcome, we constructed hierarchical logistic regression models: Model 0 (null), Model 1 (medications only), Model 2 (medications plus interactions), and Model 3 (full model with clinical covariates including age, EF, BMI, comorbidities, smoking status, and previous hospitalizations). We assessed model fit using deviance, Akaike Information Criterion (AIC), Bayesian Information Criterion (BIC), and Hosmer-Lemeshow tests, and calculated multiple pseudo R-squared measures. We examined dose-response relationships and visualized significant interactions through predicted probability plots. Statistical significance was defined as p<0.05; p-values between 0.05-0.10 were reported as non-significant. 

All statistical analyses were performed using Stata version 19 (StataCorp LLC, College Station, TX, US). 

## Results

Baseline characteristics

Among the 283 patients included in the final analysis, 141 (50%) experienced 30-day readmission, 161 (57%) experienced 90-day readmission, and 65 (23%) died within one year of the index hospitalization. The cohort demonstrated a high prevalence of cardiovascular comorbidities, with hypertension (266/283, 94%), chronic kidney disease (195/283, 69%), diabetes mellitus (184/283, 65%), and coronary artery disease (167/283, 59%) being most common (Table 1). 

**Table 1:** Baseline patient characteristics (n=283) BMI, body mass index; CHF, congestive heart failure; HFrEF, heart failure with reduced ejection fraction; HFmrEF, heart failure with mid-range ejection fraction; HFpEF, heart failure with preserved ejection fraction; NYHA, New York Heart Association.

Characteristic	n (%)
Age, years	
18 to 49	40 (14%)
50-59	57 (20%)
60-69	74 (26%)
70-79	59 (21%)
≥80	53 (19%)
CHF type	
HFrEF	102 (36%)
HFmrEF	40 (14%)
HFpEF	141 (50%)
NYHA functional class	
I	3 (1%)
II	62 (22%)
III	192 (68%)
IV	26 (9%)
Ejection fraction	
<40%	93 (33%)
40-54%	59 (21%)
≥55%	131 (46%)
Comorbidities	
Hypertension	266 (94%)
Coronary artery disease	167 (59%)
Diabetes mellitus	184 (65%)
Chronic kidney disease	195 (69%)
Previous hospitalization	235 (83%)
BMI category	
Underweight (<18.5)	8 (3%)
Normal (18.5-24.9)	48 (17%)
Overweight (25-29.9)	48 (17%)
Obese (≥30)	179 (63%)
Smoking status	
Never	161 (57%)
Former	85 (30%)
Current	37 (13%)

The majority of patients (235/283, 83%) had been previously hospitalized for HF.

With respect to HF classification, 102 (36%) patients had HFrEF, 40 (14%) had HFmrEF, and 144 (51%) had HFpEF. Most patients (192/283, 68%) were classified as NYHA functional class III, with 62 (22%) in class II, 23 (8%) in class IV, and only three (1%) in class I. Obesity was prevalent in this cohort, with 175 patients (62%) having a BMI ≥30 kg/m².

Medication utilization patterns

An analysis of GDMT utilization revealed variable prescription patterns across the four key medication classes. RAS inhibitors (ACE inhibitors, ARBs, or ARNI) were not prescribed or contraindicated in 147 patients (52%) with 79 (28%) receiving low doses, and 28 (10%) each receiving medium or goal doses. Beta blockers showed higher utilization, with 147 patients (52%) patients on low doses, 54 (19%) not receiving them, 48 (17%) on medium doses, and 37 (13%) on goal doses. MRA utilization was limited, with 226 patients (80%) not receiving this medication class. SGLT2 inhibitors had the lowest utilization at 31 patients (11%).

Predictors of 30-day readmission

Multivariable logistic regression analysis identified several significant predictors of 30-day readmission (Table 2).

**Table 2:** Multivariable predictors of 30-day readmission Wald χ², Wald chi-square test statistic; Reference category, Not receiving medication; RAS, Renin-angiotensin system; MRA, Mineralocorticoid receptor antagonist; BB, Beta blocker; GDMT, Guideline-directed medical therapy composite score (range 0-9). Results from multivariable logistic regression adjusted for age, BMI, hypertension, coronary artery disease, chronic kidney disease, and smoking status. Statistical significance defined as p<0.05. The asterisk (*) denotes categorical medication dose variables that are compared to the reference category of patients not receiving that specific medication.

Variable	Odds ratio	95% CI	Wald χ²	p-value
Medication interactions				
RAS inhibitor × Beta blocker	0.7	0.50-0.98	4.21	0.04
RAS inhibitor × MRA	0.72	0.49-1.04	3.09	0.08
Beta blocker × MRA	1.47	0.96-2.26	3.25	0.07
Categorical medication variables				
Beta blocker (low dose)*	3.34	1.44-7.74	7.89	0.01
Beta blocker (medium dose)*	3.92	1.39-11.07	6.58	0.01
MRA (low dose)*	0.27	0.11-0.64	8.94	<0.01
Medication combinations				
Combo 1 (goal dose of RAS or beta blocker)	0.41	0.19-0.91	4.82	0.03
GDMT score (per 1-point increase)	0.85	0.74-0.98	5.29	0.02
Clinical variables				
Ejection fraction (per 1% increase)	0.95	0.93-0.98	10.24	<0.01
Previous hospitalizations	3.37	1.50-7.54	8.51	<0.01
Diabetes mellitus	1.64	0.91-2.95	2.71	0.1

**Figure 1:**
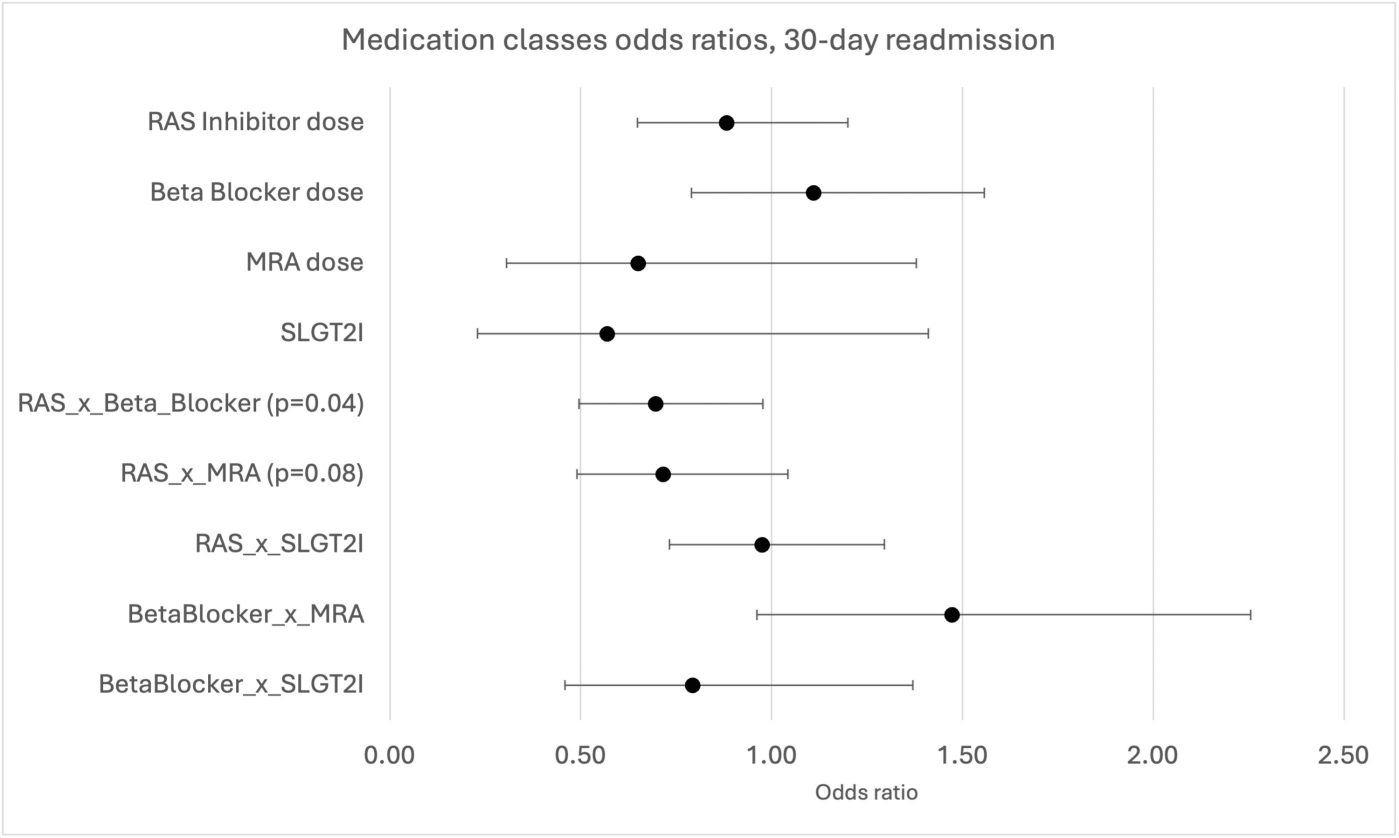
Odds ratios (ORs) for 30-day readmission across medication classes RAS, Renin-angiotensin system; MRA, mineralocorticoid receptor antagonists; SGLT2i, Sodium-glucose cotransporter 2 inhibitors. The figure illustrates the ORs for 30-day hospital readmission across medication classes and their interactions. The X-axis shows ORs (0.0 to 2.5), with 1.0 as the reference line for no effect. The Y-axis lists: RAS inhibitor dose, Beta Blocker dose, MRA dose, SGLT2i, RAS × Beta Blocker (p=0.04), RAS × MRA (p=0.08), RAS × SGLT2i, Beta Blocker × MRA, and Beta Blocker × SGLT2i. Each point marks the OR, with the horizontal bars representing confidence intervals. Most ratios are near 1.0, indicating little effect on readmission risk. The RAS × Beta Blocker interaction (p=0.04) and RAS × MRA (p=0.08) show slight deviations, suggesting potential impacts with marginal statistical significance.

When analyzing medication classes as continuous variables, no individual medication class was significantly associated with 30-day readmission (Figure 1).

**Figure 2:**
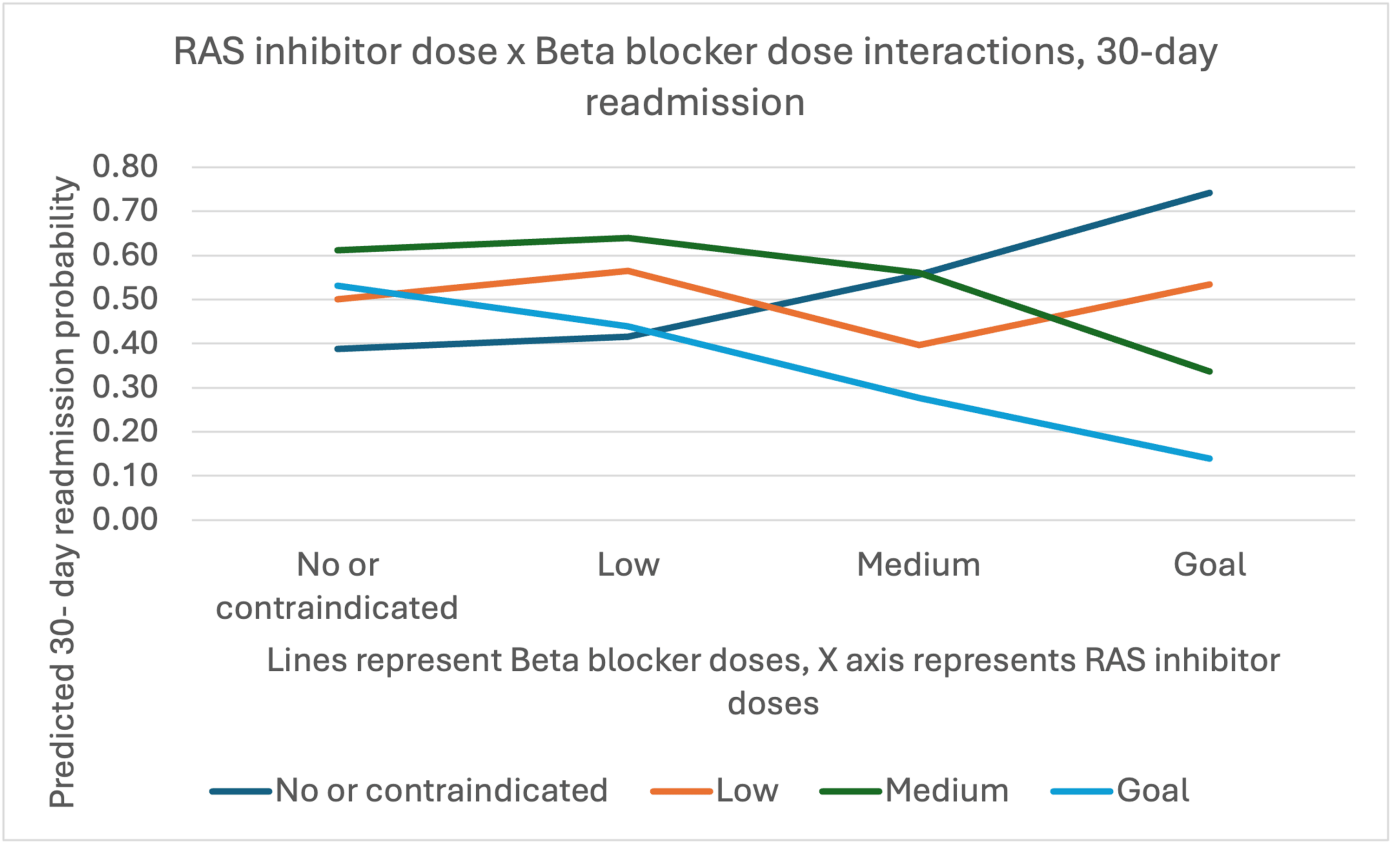
RAS inhibitor vs. beta blocker dose interaction RAS, Renin-angiotensin system. Without beta blockers, higher RAS inhibitor doses increase 30-day readmission risk. At goal beta blocker doses, higher RAS inhibitor doses reduce readmission risk. Low beta blocker doses show no significant readmission change with RAS inhibitor dose. Medium beta blocker doses lower readmission risk only at goal RAS inhibitor dose levels.

However, significant interactions were observed between medication classes. The interaction between RAS inhibitor and beta blocker doses showed statistical significance (OR=0.70, 95% CI: 0.50-0.98, p=0.04), indicating that concurrent optimization of these medications affected readmission risk (Figure 2).

**Figure 3:**
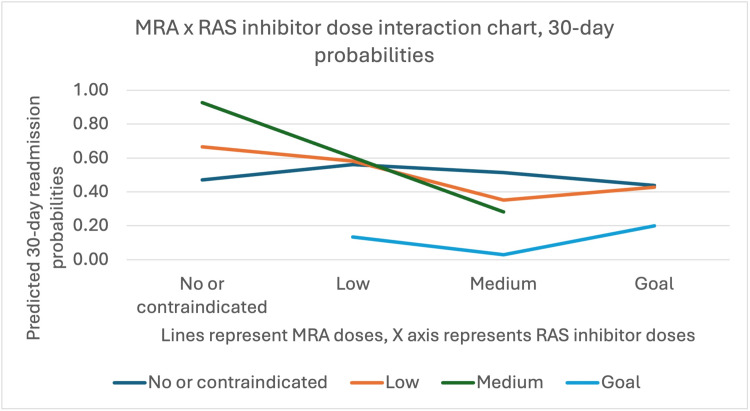
MRA vs. RAS inhibitor dose interaction chart with 30-day probabilities MRA, Mineralocorticoid receptor antagonist; RAS, Renin-angiotensin system. Without RAS inhibitors, increasing MRA dose from none to medium raises 30-day readmission risk. At medium or goal RAS inhibitor doses, higher MRA doses reduce readmission risk. At low RAS inhibitor doses, goal MRA dose levels significantly lower readmission risk compared to lower MRA doses.

Interactions between RAS inhibitor and MRA (OR=0.72, 95% CI: 0.49-1.04, p=0.08) and between beta blocker and MRA (OR=1.47, 95% CI: 0.96-2.26, p=0.07) approached but did not reach statistical significance at the p<0.05 threshold (Figure 3).

**Figure 4:**
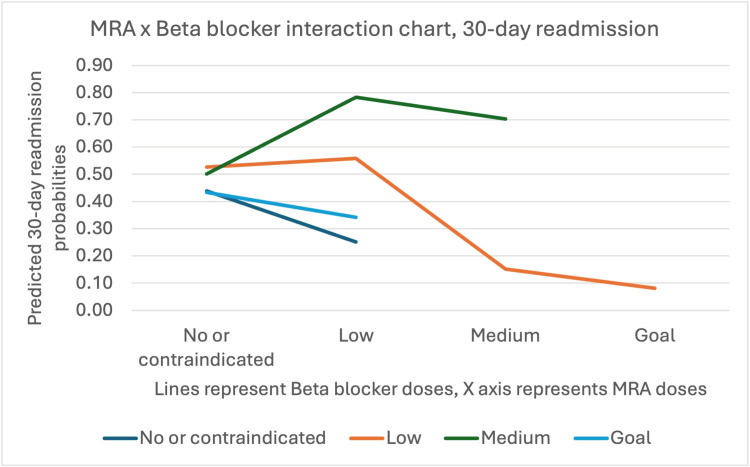
MRA vs. Beta blocker interaction chart with 30-day readmission probabilities MRA, Mineralocorticoid receptor antagonist. The MRA x beta blocker interaction chart shows that when MRA dose is 0, predicted probabilities of 30-day readmission are similar across all beta blocker doses. When MRA dose is low, predicted probabilities of 30-day readmission are markedly lower when no beta blockers are given compared to when beta blockers are given at any dose level.

When medications were analyzed as categorical variables, patients receiving low doses (OR=3.34, 95% CI: 1.44-7.74, p=0.01) or medium doses (OR=3.92, 95% CI: 1.39-11.07, p=0.01) of beta blockers had significantly higher odds of 30-day readmission compared to those not receiving beta blockers. Conversely, patients receiving low doses of MRA demonstrated lower odds of 30-day readmission (OR=0.27, 95% CI: 0.11-0.64, p<0.01) (Figure 4).

Analysis of medication combinations revealed that Combo 1 (either RAS inhibitor or beta blocker at goal dose) was associated with significantly reduced odds of 30-day readmission (OR=0.41, 95% CI: 0.19-0.91, p=0.03). Higher GDMT scores, representing greater overall guideline-directed therapy optimization, were also associated with reduced 30-day readmission risk (OR=0.85, 95% CI: 0.74-0.98, p=0.02).

Among clinical variables, lower EF (OR=0.95, 95% CI: 0.93-0.98, p<0.01) and previous hospitalizations (OR=3.37, 95% CI: 1.50-7.54, p<0.01) were significant predictors of increased 30-day readmission risk. Diabetes mellitus approached but did not reach statistical significance (OR=1.64, 95% CI: 0.91-2.95, p=0.10).

The predicted probability of 30-day readmission based on the interaction between RAS inhibitor and beta blocker doses showed that when patients received no beta blockers, the likelihood of 30-day readmission increased with increasing RAS inhibitor doses (Figure 1). However, when beta blocker doses were at goal levels, increasing RAS inhibitor dose decreased the probability of 30-day readmission. 

Predictors of 90-day readmission

For 90-day readmission, the interaction between RAS inhibitor and MRA doses approached statistical significance (OR=0.70, 95% CI: 0.47-1.03, p=0.07), as did the interaction between beta blocker and SGLT2 inhibitor (OR=0.69, 95% CI: 0.46-1.04, p=0.08). However, these interactions did not reach the conventional significance threshold of p<0.05.

**Figure 5:**
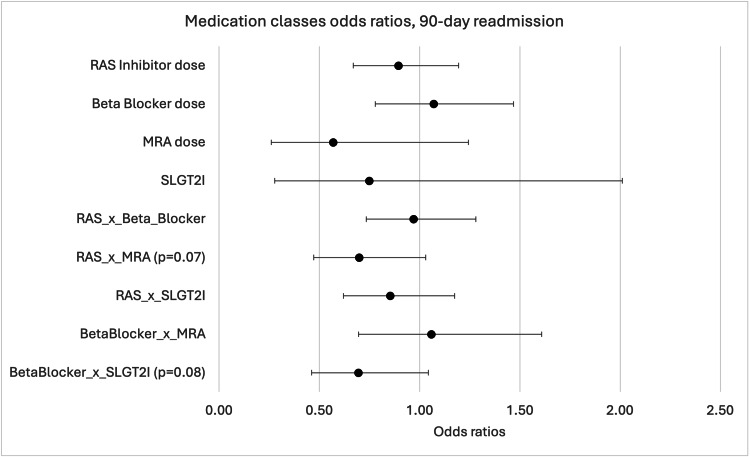
Odds ratios (ORs) for 90-day readmission across medication classes RAS, Renin-angiotensin system; MRA, mineralocorticoid receptor antagonist; SGLT2i, Sodium-glucose cotransporter 2 inhibitor. This figure shows ORs for 90-day hospital readmission across medication classes (RAS inhibitors, beta blockers, MRA, SGLT2i) and their interactions (RAS × Beta Blocker, RAS × MRA, RAS × SGLT2i, Beta Blocker × MRA, Beta Blocker × SGLT2i). Points represent ORs, with 95% CIs ranging from 0.5 to 2.0. OR<1.0 indicates reduced risk and OR>1.0 indicates increased risk. The line at OR=1.0 shows no effect. RAS × MRA (p=0.07) and Beta blocker × SGLT2i (p=0.08) interactions are near significance.

When analyzed as categorical variables, patients on low-dose beta blockers had increased 90-day readmission risk (OR=2.15, 95% CI: 1.02-4.53, p=0.04) compared to those not receiving beta blockers. Conversely, both low-dose MRA (OR=0.39, 95% CI: 0.17-0.92, p=0.03) and goal-dose MRA (OR=0.08, 95% CI: 0.01-0.74, p=0.03) were associated with significantly lower 90-day readmission risk (Figure 5).

Unlike with 30-day readmission, medication combinations (Combo 1 and Combo 2) were not statistically significant predictors of 90-day readmission. However, higher GDMT composites were associated with lower 90-day readmission risk (OR=0.86, 95% CI: 0.75-0.99, p=0.03). 

Similar to 30-day readmission, lower EF (OR=0.97, 95% CI: 0.95-1.00, p=0.02) and previous hospitalizations (OR=2.96, 95% CI: 1.44-6.08, p<0.01) remained significant predictors of increased 90-day readmission risk.

Predictors of one-year mortality

One-year mortality demonstrated different predictor patterns compared to readmission outcomes (Table 3).

**Table 3:** Multivariable predictors of one-year mortality RAS, Renin angiotensin system; MRA, Mineralocorticoid receptor antagonist; SGLT2i, sodium-glucose cotransporter 2 inhibitor; GDMT, Guideline-directed medical therapy composite score (range 0-9). Results from multivariable logistic regression adjusted for age, ejection fraction, BMI, coronary artery disease, diabetes mellitus, and smoking status. Wald χ² = Wald chi-square test statistic. Statistical significance defined as p<0.05. Reference category, not receiving medication. The asterisk (*) denotes categorical medication dose variables that are compared to the reference category of patients not receiving that specific medication.

Variable	Odds ratio	95% CI	Wald χ²	p-value
Medication variables				
Beta blocker (medium dose)*	0.13	0.02-0.79	4.89	0.03
Beta blocker (goal dose)*	0.27	0.07-1.07	3.46	0.06
MRA (medium dose)*	<0.01	<0.01-0.01	12.34	<0.01
Medication interactions				
Beta blocker × SGLT2 inhibitor	1.49	1.14-1.96	8.47	<0.01
Medication combinations				
Combo 2 (medium dose of beta blocker, RAS, or MRA)	0.43	0.19-0.98	4.01	0.04
GDMT score (per 1-point increase)	0.93	0.78-1.11	0.61	0.42
Clinical variables				
Hypertension	0.3	0.10-0.93	4.32	0.04
Chronic kidney disease	2.49	1.12-5.50	4.97	0.03
Previous hospitalizations	3.96	1.33-11.76	6.12	0.01

When analyzed as continuous variables, both higher beta blocker doses (OR=0.63, 95% CI: 0.43-0.93, p=0.02) and higher MRA doses (OR=0.34, 95% CI: 0.13-0.92, p=0.03) were independently associated with lower mortality risk. Additionally, the interaction between beta blocker and SGLT2 inhibitor was statistically significant (OR=1.49, 95% CI: 1.14-1.96, p<0.01).

In the categorical analysis, medium-dose beta blockers demonstrated a strong protective effect against one-year mortality (OR=0.13, 95% CI: 0.02-0.79, p=0.03), as did medium doses of MRA (OR<0.01, p<0.01). Goal doses of beta blockers approached statistical significance for mortality reduction (OR=0.27, 95% CI: 0.07-1.07, p=0.06).

Medication Combo 2 (at least one of beta blocker, RAS inhibitor, or MRA at medium dose) was associated with a significantly lower one-year mortality (OR=0.43, 95% CI: 0.19-0.98, p=0.04). Interestingly, GDMT scores were not statistically significant predictors of one-year mortality after adjustment for confounding variables, although they approached significance in the unadjusted model (OR=0.86, 95% CI: 0.74-1.01, p=0.07).

**Figure 6:**
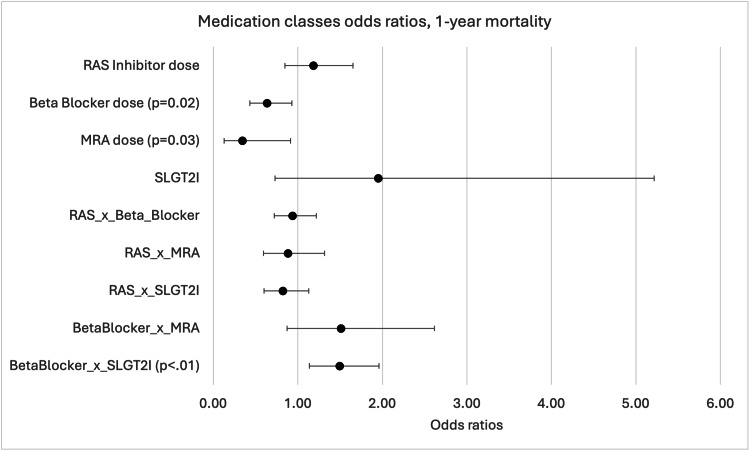
Odds ratios (ORs) for one-year mortality across medication classes RAS, Renin-angiotensin system; MRA, mineralocorticoid receptor antagonist; SGLT2i, Sodium-glucose cotransporter 2 inhibitor. This forest plot displays ORs for one-year mortality across various medication classes. Beta blocker dose (p=0.02), MRA dose (p=0.03), and Beta blocker_x_SLGT2i (p<0.01) showed statistically significant reductions in mortality, with OR<1. RAS inhibitor dose, SLGT2i, RAS_x_Beta_blocker, RAS_x_MRA, and RAS_x_SLGT2i showed no significant effect, with ORs close to 1. CIs were narrow, indicating precise estimates.

Among clinical variables, the absence of hypertension (OR=0.30, 95% CI: 0.10-0.93, p=0.04), presence of chronic kidney disease (OR=2.49, 95% CI: 1.12-5.50, p=0.03), and previous hospitalizations (OR=3.96, 95% CI: 1.33-11.76, p=0.01) were significant predictors of increased one-year mortality (Figure 6).

Dose-response relationships

An examination of the dose-response relationships between medication classes and outcomes revealed generally linear trends for one-year mortality with some exceptions for GDMT scores. For 30-day and 90-day readmissions, notable deviations from linearity were observed at low and medium levels of RAS inhibitors and beta blockers, suggesting complex relationships between medication dosing and readmission risk that may not be adequately captured by linear models.

The strongest dose-response relationship was observed for beta blockers and one-year mortality, with increasing doses associated with progressively lower mortality risk. Medium and goal doses of MRA also showed substantial mortality benefit compared to no MRA, though the relationship was not perfectly linear across all dose categories.

GDMT distribution and analysis

In our cohort, GDMT composites exhibited a right-skewed distribution with a mean of 2.4±1.8 and median of 2.0 (IQR: 1.0-3.0). Only 8% of patients achieved a GDMT score of five or higher, indicating a substantial opportunity for medication optimization in this population.

The most common GDMT score was one (28% of patients), followed by scores of two (26%) and three (18%), respectively. Fourteen percent of patients had a GDMT score of zero, indicating no prescribed HF medications out of the four guideline-recommended classes. At the other end of the spectrum, only 2% of patients achieved GDMT scores of eight or higher, representing near-optimal guideline-directed therapy.

**Figure 7:**
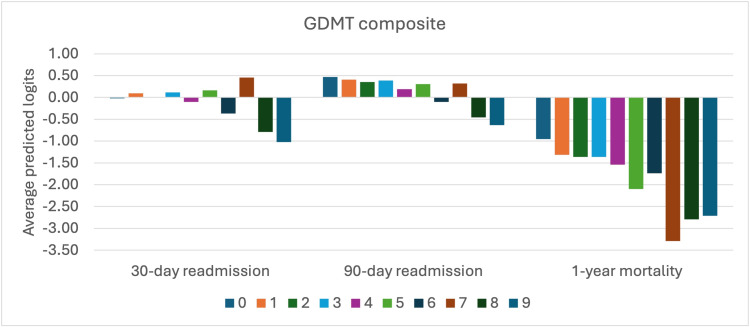
Guideline-directed medical therapy composite This bar chart illustrates the average predicted logits for the three clinical outcomes: 30-day readmission, 90-day readmission, and one-year mortality, across nine categories (zero to nine, each uniquely colored). The Y-axis spans average predicted logits from -3.50 to 1.00. For 30-day readmission, logits are near zero, with category zero being slightly positive. For 90-day readmission, categories zero and two show negative logits around -0.50. For one-year mortality, all categories have negative logits, with category seven at -3.50, indicating a strong negative prediction. The chart reveals distinct predictive patterns across the outcomes and categories.

When stratified by HF type, patients with HFrEF had significantly higher mean GDMT scores (3.1±2.0) compared to those with HFmrEF (2.2±1.6) or HFpEF (1.9±1.5) (p<0.001), consistent with stronger evidence and guideline recommendations for medication therapy in HFrEF (Figure 7).

In multivariable analysis, each one-point increase in GDMT score was associated with a 15% reduction in 30-day readmission risk (OR=0.85, 95% CI: 0.74-0.98, p=0.02) and a 14% reduction in 90-day readmission risk (OR=0.86, 95% CI: 0.75-0.99, p=0.03). For one-year mortality, the GDMT score approached significance in the unadjusted model (OR=0.86, 95% CI: 0.74-1.01, p=0.07) but was not significant after adjustment for clinical covariates (OR=0.93, 95% CI: 0.78-1.11, p=0.42).

The relationship between GDMT scores and predicted probabilities of each outcome demonstrated distinct patterns. For readmission outcomes, a nearly linear relationship was observed, with each incremental increase in GDMT score corresponding to progressively lower readmission risk. For mortality, the relationship was more complex, with an initial decrease in mortality risk as GDMT composite increased from zero to three, followed by a plateau between scores of three and six, and then a further decrease at higher scores. This non-linear pattern suggests that the relationship between medication optimization and mortality may be threshold-dependent rather than strictly dose-dependent.

When examining the contribution of individual medication classes to the GDMT composite, beta blockers contributed the most to the average score (mean contribution: 1.2 points), followed by RAS inhibitors (0.8 points), SGLT2 inhibitors (0.3 points), and MRAs (0.2 points). This distribution reflects both the relative frequency of prescription and the dosing patterns of each medication class in the study population.

The strong association between GDMT composites and readmission outcomes, even after adjustment for clinical variables, supports the importance of comprehensive medication optimization in reducing HF readmissions. The less robust association with mortality suggests that while medication optimization is necessary for improving survival, other factors may play more dominant roles in determining mortality risk in this population. 

## Discussion

Our study examined the relationship between GDMT and clinical outcomes among African-American female patients with HF in a safety-net hospital setting. We found associations between incremental improvements in medication optimization and reduced hospital readmissions, with different medication classes showing varying associations with readmission versus mortality outcomes.

GDMT utilization and dosing patterns

The low rates of optimal medication dosing observed in our cohort, one in 10 patients for RAS inhibitors and beta blockers, align with patterns reported in national registries. MRA and SGLT2 inhibitor utilization was even lower at one-fifth and one-tenth of patients, respectively. This extends observations from larger studies, like CHAMP-HF, to a specific population of African-American female patients in a safety-net hospital setting [13].

The particularly low adoption of newer therapy components may reflect multiple factors, including their recent addition to guidelines and limited representation of our study population in pivotal trials. However, without directly measuring prescribing decisions, we cannot determine the specific reasons for these utilization patterns. Future studies examining factors influencing medication selection and dosing in safety-net hospital settings would help clarify these patterns.

Clinical outcomes and medication effectiveness

Each unit increase in our GDMT composite score was associated with a 15% reduction in 30-day readmission risk and a 14% reduction in 90-day readmission risk. The observation that RAS inhibitors and beta blockers together were associated with greater readmission reduction than either alone suggests potential synergistic effects, though our observational design cannot establish causation.

Medium-dose therapy, particularly for beta blockers and MRAs, showed associations with reduced mortality. Even low-dose MRA therapy was associated with fewer readmissions. These associations suggest that partial optimization may provide clinical benefit, though prospective studies would be needed to confirm these relationships.

The strong association between previous hospitalizations and subsequent readmissions identifies a potentially high-risk period. Whether enhanced monitoring during this period would improve outcomes requires prospective evaluation.

Study limitations

Our single-center, retrospective design limits both generalizability and the ability to determine causation. The observational nature of the study means that medication prescription patterns likely reflect unmeasured patient characteristics that may also influence outcomes. We lacked data on medication adherence, reasons for specific dosing decisions, and social factors that might influence both treatment and outcomes.

The absence of a comparison group and the possibility of selection bias further limit interpretation. Patients receiving higher doses of medications may have been healthier or more engaged in their care in ways we could not measure.

Future directions

Prospective studies are needed to determine whether systematic approaches to medication optimization can improve outcomes in safety-net populations. Research examining optimal sequencing strategies for patients unable to tolerate full GDMT would provide valuable guidance. Studies directly measuring barriers to medication optimization and testing interventions to address them would help translate these observational findings into actionable strategies.

## Conclusions

In this observational study of African-American female patients with HF in a safety-net hospital, we found associations between medication optimization and improved clinical outcomes. Higher GDMT composite scores were associated with fewer readmissions, while specific medications showed different patterns of association with readmission vs. mortality. These findings suggest that even partial medication optimization may be associated with a clinical benefit, though prospective studies are needed to establish causal relationships and guide implementation strategies. Our results provide preliminary evidence that strategic medication selection may be important when complete optimization is not achieved; however, confirmatory studies in diverse populations are warranted.
